# Cyto-architecture of *Byblis* glands and leaf cells based on freeze-substitution and conventional TEM

**DOI:** 10.1093/aob/mcae173

**Published:** 2024-09-27

**Authors:** Bartosz J Płachno, Sue Lancelle, Piotr Świątek, Peter K Hepler, Marieluise Weidinger, Irene Lichtscheidl

**Affiliations:** Department of Plant Cytology and Embryology, Institute of Botany, Faculty of Biology, Jagiellonian University in Kraków, 30-387 Kraków, Poland; Biology Department, University of Massachusetts Amherst, 221 Morrill Science Center III, Amherst, MA 01003-9297, USA; Institute of Biology, Biotechnology and Environmental Protection, Faculty of Natural Sciences, University of Silesia in Katowice, 40-007 Katowice, Poland; Biology Department, University of Massachusetts Amherst, 221 Morrill Science Center III, Amherst, MA 01003-9297, USA; Cell Imaging and Ultrastructure Research, University of Vienna, A-1090 Vienna, Austria; Cell Imaging and Ultrastructure Research, University of Vienna, A-1090 Vienna, Austria

**Keywords:** Carnivorous plants, cryopreservation, high-pressure freezing, freeze-substitution, organelle interactions, plant trichome, transfer cells, wall ingrowths, ultrastructure, electron microscopy

## Abstract

**Background and Aims:**

*Byblis liniflora* (Byblidaceae) is a carnivorous plant that has developed sticky flypaper traps with two types of glandular trichomes producing digestive enzymes and sticky mucilage. This study aimed to analyse the ultrastructure of these glandular leaf trichomes based on rapid freeze-fixation and conventional chemical fixation in the attempt to understand their functional contribution to the carnivorous performance of the plants.

**Methods:**

The *Byblis* cells were studied in transmission electron microscopy, scanning electron microscopy and scanning transmission electron microscopy using cryo-techniques for fixation and substitution in addition to conventional chemical fixation.

**Key Results:**

We show in detail the architecture of both the digestive glands and the mucilage glands with their relevant sets of organelles. Both mitochondria and plastids have a conspicuous plasticity, with branches and constrictions, and they associate to form clusters. The glandular cells appear to be transfer cells with cell wall ingrowths. Digestive glands occur in different states of development. Their cuticle forms discontinuities that are unique among glands of carnivorous plants. They look like cuticular holes – the cuticle separates from the cell wall in only one spot and then ruptures. Cuticular discontinuities thus differ from the cuticular gaps and cuticular pores so far described in carnivorous plants. We therefore propose for them the term ‘cuticular holes’.

**Conclusions:**

Application of cryo-techniques made it possible to show the true structure of the cell wall and the relationship between cell wall ingrowths and organelles, as well as the morphology and structure of organelles and their associations.

## INTRODUCTION

In the study of cells, it is important to get reliable results on cell structure as well as activity. Of course, it is best to study cells in their living state (Lang *et al.*, 1995; Sheen *et al*., 1995; Lichtscheidl and Foissner, 1996; Lichtscheidl and Baluška, 2000; Volgger *et al*., 2010), but this is not always possible and these techniques sometimes do not capture all details of the structure. In electron microscopy, one of the critical requirements for studying cell ultrastructure is good-quality fixation. Traditional fixation with formaldehyde and glutaraldehyde may, for certain cells or tissues, be too slow and allow rearrangement of cytoplasm and organelles (Mersey and McCully, 1978; McCully and Canny, 1985). However, everything depends on the type of tissue or species, as some authors have obtained very good results using chemical fixation (e.g. Vassilyev, 2005; Muravnik *et al*., 2016, 2019). Cryofixation can solve the problem of slow fixation because it preserves the cytoplasm in a fraction of a second (e.g. Studer *et al*., 2001; Kuo, 2007; Karahara and Kang, 2014; Gergely *et al*., 2018; Boulogne *et al*., 2020); however, the formation of ice crystals can be a problem. Therefore, it is useful to use different methods to get a comprehensive picture.

The carnivorous plants of the genus *Byblis* (rainbow plants, Byblidaceae, Lamiales, eight recognized species) are herbs or herbaceous subshrubs native to Australia and New Guinea (Lowrie and Conran, 1998, 2007; Conran *et al*., 2002; Lowrie, 2013; Cross *et al*., 2018). They all form sticky flypaper traps from slender cylindrical leaves that are studded all over with capitate glandular trichomes. They attract, retain and digest animals, mainly small insects, and absorb the nutrients from their prey. This carnivorous character of the Byblidaceae has evolved within the Lamiales independently (Cross *et al*., 2018; Fleck and Jobson, 2023), in parallel with their sister clade of Lentibulariaceae, where the boreotropical genus *Pinguicula* has developed very similar carnivorous strategies and morphologically similar glands; some doubts remain, however, about the phylogenetic position of the *Byblis* genus (Fleck and Jobson, 2023).

**Fig. 1.**
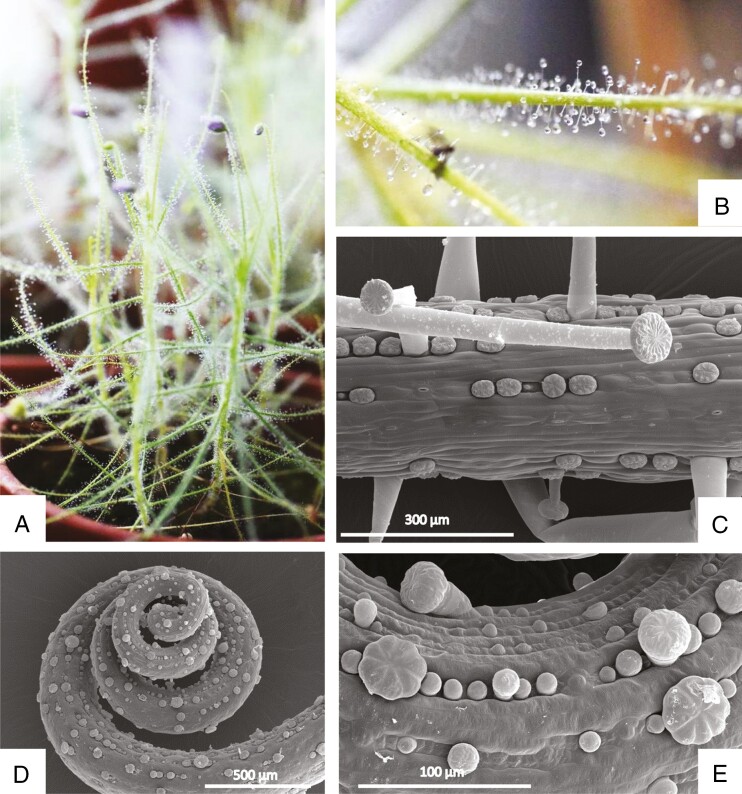
General morphology of *Byblis liniflora*. (A) Cultivated plant. (B) Macro of the leaf surface. Notice the small fly caught by the plant. (C–E) SEM of the leaf surface. (C) Mature leaf with two types of trichome: mature and developing sessile trichomes and mucilage producing trichomes with different length of stalk cell. (D) Coiled apex of the developing leaf. (E) Higher magnification of the developing leaf showing various stages of trichome development.

The leaves of *Byblis liniflora* (in contrast to *B. gigantea*, Juniper *et al*., 1989, p. 52) develop in a reverse circinate pattern from an apical meristem. Leaves, stems, flower pedicles and even sepals are covered by capitate trichomes of two types of glands, i.e. stalked trichomes producing sticky trapping mucilage that forms a glistening droplet at the tips of the glandular hairs in order to attract and retain insects, and small sessile trichomes producing digestive enzymes (e.g. Lang, 1901; Fenner, 1904; Lloyd, 1942; Płachno *et al*., 2006; Adlassnig *et al*., 2010) (Fig. 1A–C). They occur on the epidermis of mature leaves in different developmental stages. In the past these traps were considered as non-motile, but Allan (2019) and recently Poppinga *et al*. (2022) showed that stalked trichomes of *Byblis* respond with slow movements after chemical (protein) stimuli.

The carnivorous character of the genus was first suggested by Darwin (1876) and was confirmed by further research (reviewed e.g. by Juniper *et al*., 1989; Ellison and Adamec, 2018). The question of whether all *Byblis* species were able to digest their prey, a prerequisite for a true carnivorous plant, remained open. While *B. filifolia* and *B. gigantea* yielded positive results in digesting gelatin of photographic film, *B. liniflora* did not (Hartmeyer, 1997; Wallace *et al*., 1999). The question was, however, answered by Płachno *et al*. (2006), who reported high phosphatase activity in the digestive glands of *B. liniflora*. In a recent report, Li *et al*. (2023) described the physiological traits of the stalked and sessile glandular trichomes of *Byblis guehoi* and proved their dual character: stalked glands produce sticky trapping mucilage, whereas sessile glands are responsible for digestion of the prey. Uptake of digested compounds occurs by endocytosis in both gland types (Li *et al*., 2023). Information is still missing about the ultrastructure of the secretory apparatus in relation to the plants’ morphology and physiology. This requires analysis by electron microscopy (EM). However, preparation for EM is a challenge because of the extensive cutinization of the cell walls of all aerial parts of the plants, especially of some cells of the trichomes; it prevents fast exchange of solutes during conventional chemical fixation. Therefore, in addition to conventional chemical fixation, we applied cryo-techniques for fixation and substitution of *Byblis* leaves and describe the cytomorphology of the different types of cells of glandular trichomes in an attempt to understand their functional contribution to the carnivorous performance of the plants. With these results, we compare and evaluate the performance of chemical versus cryofixation preparation techniques for transmission electron microscopy (TEM). In addition, the results provide the basis for further comprehension of the phylogenetic position of the *Byblis* genus (Fleck and Jobson, 2023).

## MATERIALS AND METHODS

### Plant material

*Byblis liniflora* grew in soil in the greenhouse of the University of Massachusetts-Amherst (1988–89) and in the greenhouse of the Botanical Garden of the Jagiellonian University (2023–24). Pieces of developing and of mature leaves were dissected and used for observation in the light microscope and for preparation for EM.

### Conventional fixation for scanning TEM

For observation by EM, the preparation of the glands was as follows. Fragments of the traps were fixed in a mixture of 2.5 % glutaraldehyde with 2.5 % formaldehyde in 0.05 m cacodylate buffer (Sigma–Aldrich, Poznań, Poland; pH 7.2) overnight or for several days; later the material was processed as in Płachno *et al.* (2017). Briefly, material was dehydrated with acetone and embedded in an Epoxy Embedding Medium Kit (Fluka). Semi-thin sections (0.9–1.0 μm thick) were prepared for light microscopy and stained for general histology using aqueous methylene blue/azure II for 1–2 min. A histochemical procedure with fixed material using the periodic acid–Schiff (PAS) reaction was performed to detect polysaccharides (Wędzony, 1996). Ultrathin sections were cut on a Leica Ultracut UCT ultramicrotome. The sections were examined using a Hitachi UHR FE-SEM SU 8010 microscope (Hitachi, Tokyo, Japan), which is housed at the University of Silesia in Katowice.

### High-pressure freeze-fixation and freeze-substitution for TEM

For freeze preparation, we followed the protocol that proved successful for the investigation of *Drosera* (Lichtscheidl *et al*., 1990, 2021). Briefly, leaf sections were placed between two lecithin-coated gold sample holders (Balzers BB1131242-1). A droplet of 1 % ultralow gelling (<1 °C) agarose (Type IX, Sigma) surrounded the tissue and served for mechanical protection as well as for maximum heat conductivity. The tissues were frozen in a Balzers HPM 010 high-pressure freezer. For freeze-substitution, we followed the protocol of Lancelle *et al*. (1986, 1987) and of Kiss *et al.* (1990) and Staehelin *et al.* (1990). Cryoimmobilized material was transferred to acetone containing 2 % osmium tetroxide at ~−80 °C and freeze-substituted for 36–40 h, then brought to room temperature gradually over a period of 5–6 h. Before embedding in a mixture of Epon and Araldite, the tissue was transferred to methanol and stained in 5 % uranyl acetate in methanol for 2 h, then brought back to acetone. After staining with lead citrate, ultrathin sections were observed in a Jeol 100 CX electron microscope (Jeol, Tokyo, Japan) operated at 80 kV.

### Observations in scanning electron microscope

For the scanning electron microscope (SEM), the traps were fixed in a mixture of 2.5 % glutaraldehyde with 2.5 % formaldehyde in 0.05 m cacodylate buffer or methanol (100 %) (at room temperature) and later transferred to ethanol (10, 30, 50, 70, 95, 100%, 5 min each step), then transferred to acetone (100 %) and dried using supercritical CO_2_ in an E3000 critical point dryer (Quorum Technologies, Laughton, UK). The material was then sputter-coated with gold (using a Jeol JFC-1100E, Jeol, Tokyo, Japan) and examined at an accelerating voltage of 20 kV using a Hitachi S-4700 SEM (Hitachi, Tokyo, Japan), which is housed at the Institute of Geological Sciences, Jagiellonian University in Kraków, Poland.

## RESULTS

Two different types of glandular trichomes nestle into lengthy indentations of the leaf surface and occur side by side in different developmental stages (Fig. 1C–E). They are in homology considering their micromorphological organization: secretory cells are arranged in a concentric circle and form a multicellular head that is supported by one endodermal cell (often also called a ‘neck cell’ or ‘neck’); it separates the glandular cells from the rest of the leaf. This glandular apparatus is carried by basal cells of the leaf either directly (sessile glands) or with a unicellular stalk of variable length that lifts the head above the surface (stalked glands). The two gland types show conspicuous differences in ultrastructure and physiology: while stalked glands are said to be permanently mucilaginous, sessile glands produce digestive enzymes only once (Juniper *et al*., 1989, p. 52). We investigated the ultrastructure of the various types of cells during the secretory phase of the mature leaves.

### Stalked trichomes produce sticky trapping mucilage

**Fig. 2.**
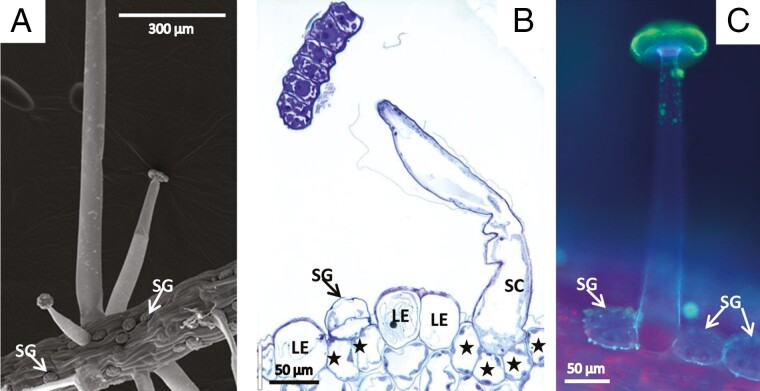
Stalked mucilage-producing glands in SEM (A), after histochemical treatment using aqueous methylene blue/azure II (B) and in fluorescence (C). (A) The head of glandular cells is carried above the surface of the leaf on a stalk cell. The stalks have different lengths, depends on the glands. Sessile glands (SG) nestle into the epidermis (arrow). (B) Basal cells (asterisk) are submerged into the leaf surface (LE, leaf epidermis) and carry the stalk cells (SC). Histochemical treatment with aqueous methylene blue for 2 min shows the difficulty of fixing all kinds of cells except for glandular cells due to the impenetrable cuticle: fixatives need a long time to reach the cytoplasm, and the turgor is lost. The arrow points to a sessile gland (SG) above two basal cells. (C) Impregnation of cell walls with cutin is shown by blue autofluorescence in UV light. The cuticle covers the whole surface of the trichome, but is especially well developed in the lateral walls of the endodermal cell. The sticky mucilage has yellow fluorescence.

**Fig. 3.**
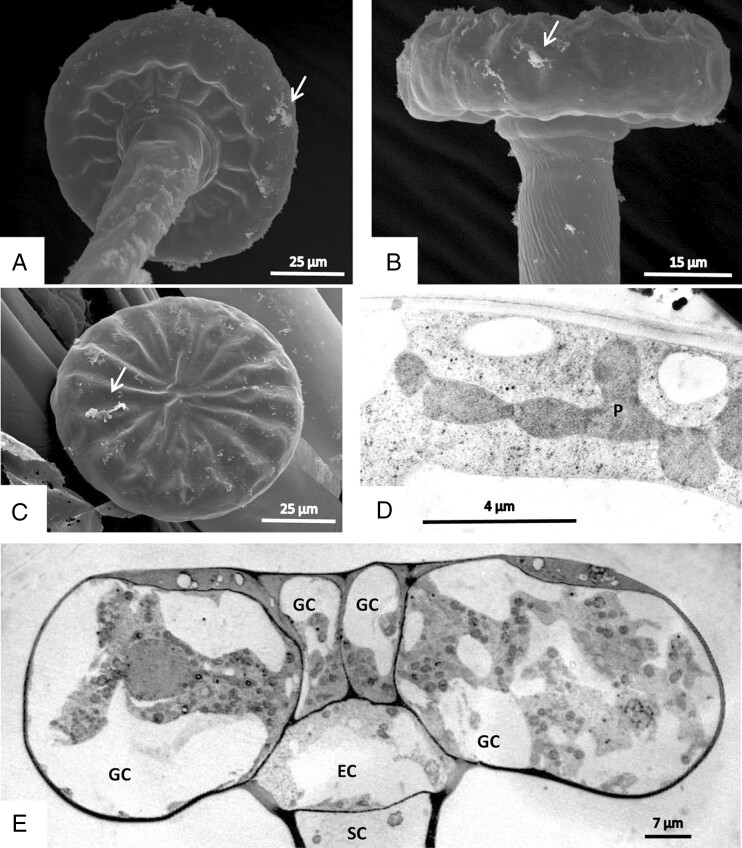
Stalked mucilage producing glands. (A–C) Glandular cells in SEM. They form a head that is supported by a unicellular stalk. Note the elevated bumps in the apical cell walls. The arrows point to smears presumably derived from secreted mucilage. (D, E) TEM after cryofixation and freeze-substitution. (D) Leucoplasts (P) in the parietal cytoplasm of the glandular cells form interconnected clusters. (E) Median vertical section shows an endodermal cell (EC) that connects the glandular cells (GC) to the stalk cell (SC). The fast fixation makes visible the fragmented vacuoles (panel E) and the clusters of leucoplasts (panel D) in the glandular cells.

Stalked trichomes consist of a group of basal cells submerged in the epidermis (Fig. 2B; on the section we observed up to four cells), a stalk cell of variable length (up to 1 mm long) that raises the head above the leaf surface (Fig. 2A), an endodermal cell and a head of up to 100 µm diameter that is formed of terminal secretory cells, usually 16–32 cells (Fig. 3A–C). The head of each stalked trichome is enveloped by a drop of sticky mucilage (Fig. 1A, B). Many glandular trichomes contain one or a few secretory cells in their head where the cytoplasm is decayed and degenerated, although the neighbouring cells look perfectly healthy. This is also visible in living plants observed in the light microscope (not shown) and therefore most probably is not a consequence of fixation but a physiological feature.

#### Secretory cells.

Mitochondria, plastids and dictyosomes together with small empty-looking vacuoles occur in the cytoplasm together with a large fragmented vacuole (Fig. 3D, E). In some glands they form clusters, especially where the nucleus lies in a cytoplasmic pocket, often in that part of the cell that connects to the endodermal cell below the glandular head.

**Fig. 4.**
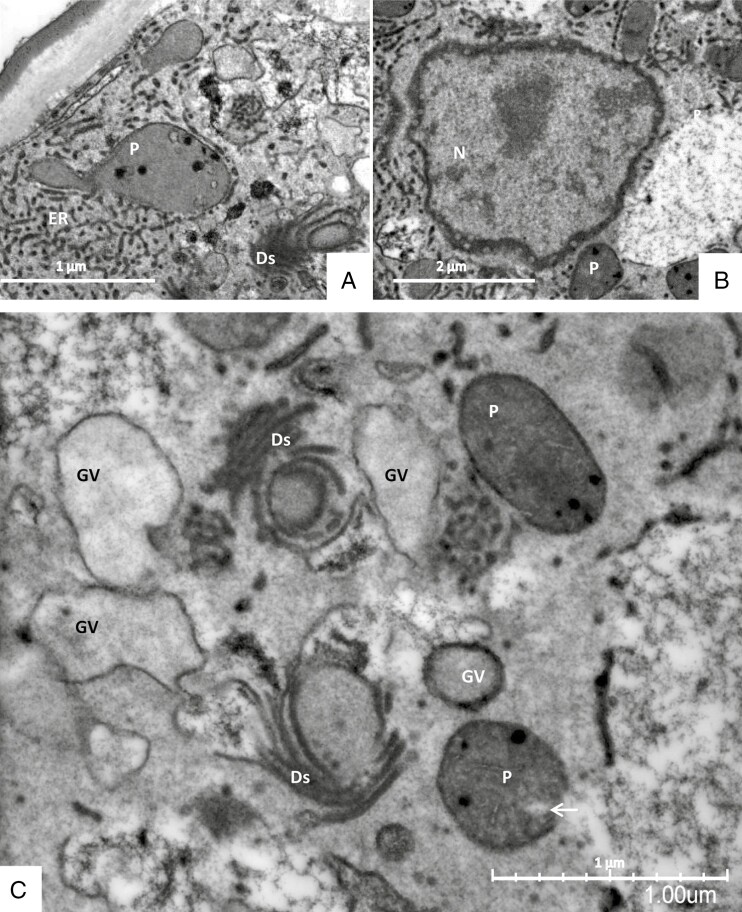
Secretory cells from stalked glands in TEM after chemical fixation (A–C). Dictyosomes (Ds) appear active and produce large darkly or lightly stained Golgi vesicles (GV). A net of darkly stained smooth tubular ER traverses the whole cytoplasm and also clusters in some areas and encloses the nucleus (N) and plastids (P). Plastids have tiny starch grains (arrow) and osmiophilic inclusions.

**Fig. 5.**
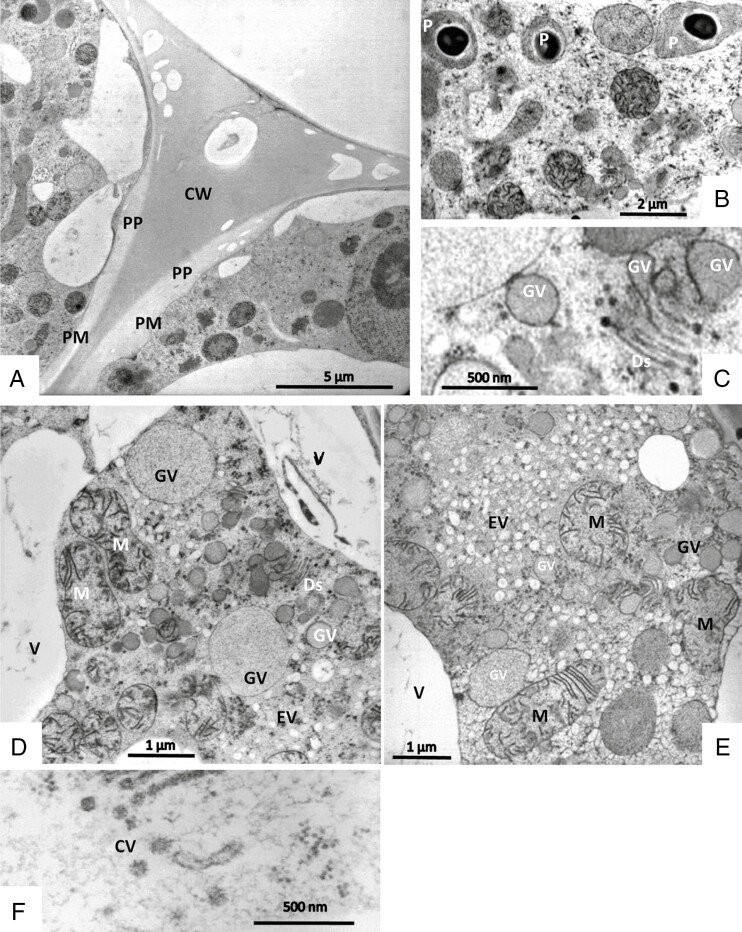
Secretory cells from mucilage-producing stalked glands in TEM after cryofixation. (A) The cell wall (CW) at the periphery contains holes. It is lined in some areas by a smooth layer of lightly stained homogeneous substance forming a periplasmic space (PP) also containing holes. The plasma membrane (PM) closely borders the cell wall and periplasmic space. (B) Leucoplasts (P) contain dark roundish osmiophilic bodies. (C) Large slime-containing Golgi vesicles (GV) are formed directly by Golgi cisternae (dictyosomes, Ds); in addition they fuse with small vesicles. (D) Mitochondria (M) have well-developed cristae. (E) The cytoplasm becomes filled with transparent empty looking vesicles (EV) in addition to the large vacuole (V). They are especially visible in freeze-fixed cells. (F) Vesicles may be coated by spikes resembling clathrin (CV coated vesicles).

The ultrastructure of organelles can be well seen after both chemical fixation (Fig. 4) and cryofixation (Fig. 5). Plastids (leucoplasts) are polymorphic; their lamellae are only poorly developed, they have small osmiophilic inclusions and small starch grains. In freeze-fixed cells, they appear as aggregates of five or more organelles that are associated in line and form branches (Fig. 3D). Mitochondria have well-developed cristae. The nucleus has a bumpy surface; on its envelope, nuclear pores are clearly visible (Fig. 4B). An intricate network of tubular endoplasmic reticulum (ER) with densely stained content and some dilated profiles traverses the whole cytoplasm and closely attaches to plastids and nucleus. Dictyosomes appear very active; they have five to eight flattened cisternae that can be curled or in stair-stepped form; they produce small and large vesicles containing a homogeneous greyish substance assumed to be the trapping mucilage. Freeze-fixation allows the visualization of fusion of these small and large slime-containing vesicles. In older cells, the large secretory vesicles lose their contact with the hypertrophic Golgi bodies; only a few small vesicles remain at their borders. Vesicles may be covered by a coat of spikes resembling clathrin (Fig. 5F). In addition, the cytoplasm becomes filled with transparent-looking vesicles, which are especially visible in freeze-fixed cells.

The walls of the secretory cells are convex at their apical distal surface, and covered by a cuticle. Pores are not visible, but in SEM we see a substance smeared over the surface that is presumably derived from secreted mucilage (Fig. 3A–C). In UV light, this secretion has green autofluorescence (Fig. 2C). The walls of freeze-fixed cells contain electron-light holes, and a lining of homogeneous lightly stained material towards the cytoplasm. This is closely aligned with the plasma membrane (Fig. 5A).

#### Endodermal cell.

**Fig. 6.**
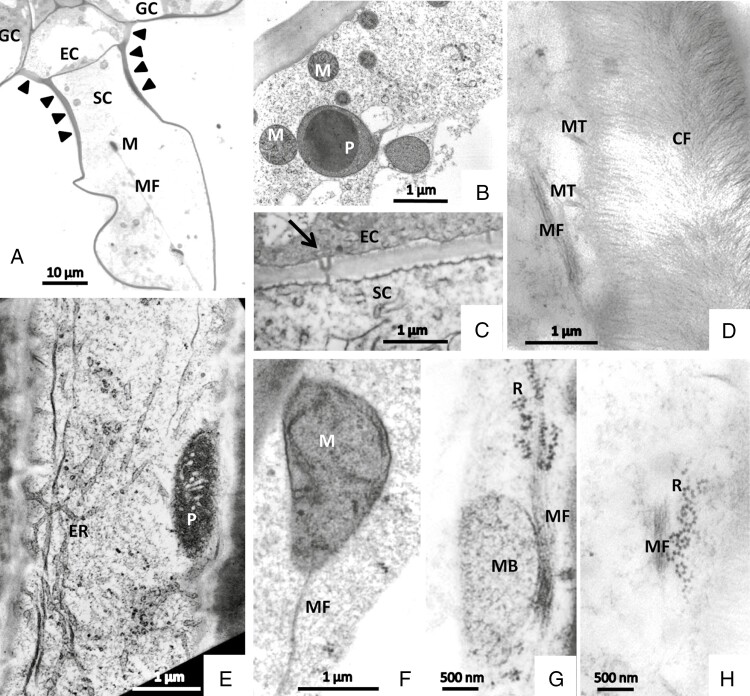
Stalked mucilage-producing glands in TEM after freeze-fixation. (A) The endodermal cell (EC) is carried by the unicellular stalk. The stalk cell (SC) is collapsed during the embedding process. The cell wall of the endodermis and of the upper part of the stalk is heavily cutinized (arrowheads). (B) The cytoplasm contains plastids (P) with large osmiophilic inclusions, and mitochondria (M). (C) Transverse wall between endodermis (EC) and stalk (SC) is perfused by plasmodesmata, which may be branched (arrow). (D–H) Details of the stalk cell. The parallel arrangement of cellulose fibrils (CF) is clearly visible (D). Cortical microtubules and bundles of actin microfilaments are well preserved (D–H). The ER forms a cortical network, and plastids attach closely to the cell wall (E). Mitochondria (M) (A, F) and microbodies (MB) (G) bind closely to microfilaments, as well as ribosomes (R) (G, H). GC = glandular cell, MF = actin microfilaments, MT = microtubules.

The endodermal cell is a barrier cell for apoplastic transport due to strong impregnation of the lateral cell walls with cutin. This cell is highly vacuolated. Plasmodesmata occur in the transverse walls between endodermal cells and secretory cells, and between endodermal cells and stalk cells; they may be branched (Fig. 6C).

The plastids contain scarce tubular membrane elements, a few starch grains, and large globular electron-opaque intraplastidial bodies.

#### Stalk cell

*.* The cylindrical stalk cell is of variable length, up to 1 mm long, and has a central vacuole that shows transvacuolar cytoplasmic strands (Fig. 6A). Organelles move within the peripheral cytoplasm and through cytoplasmic strands with velocities up to 8 µm s^−1^, as measured by observation in the light microscope. This relates to the well-preserved cytoskeleton consisting of microtubules and bundles of actin microfilaments in freeze-fixed cells (Fig. 6D, F–H). In addition, the cytoplasm contains a well-developed network of rough ER, mitochondria, plastids with small starch grains and dictyosomes. The cortical ER coaligns with mitochondria and plastids, and coated vesicles from the trans-Golgi network are closely associated with organelles such as mitochondria (Supplementary Data Fig. 1).

Cell walls are cutinized and we observed that both cell wall and cutinization are especially strong underneath the endodermal cell (Figs 2 and 6). Plasmodesmata occur in the transverse periclinal cell walls between various gland cells. Within the cell wall, the parallel cellulose microfibrils are organized angular to the long axis of the cell. During chemical fixation and also during cryo-substitution after freeze-fixation, the stalk cells collapse; either their walls fold on each other or the whole of the long cell twists, makes wrinkles and folds.

### Sessile trichomes produce digestive enzymes

**Fig. 7.**
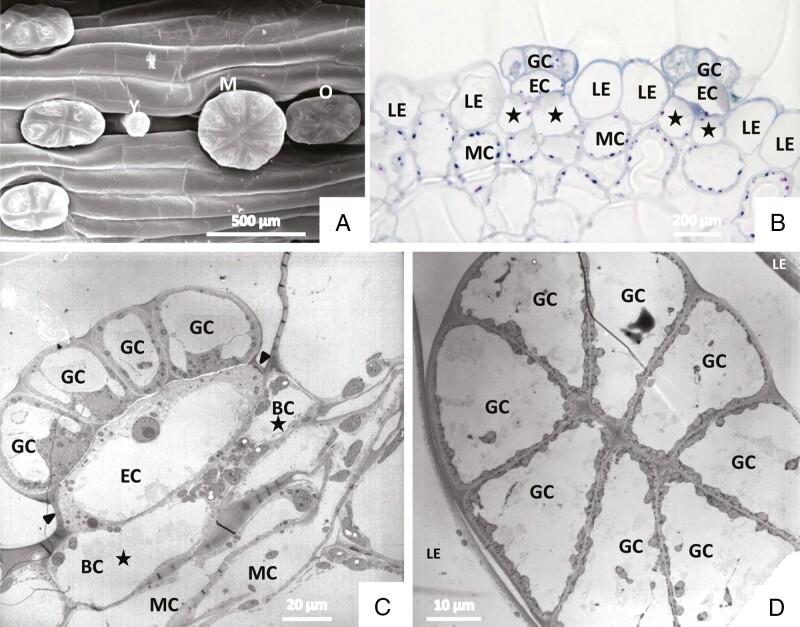
Sessile glandular trichomes. (A) SEM of glands of different developmental states. Y, young gland; M mature gland; O old gland, collapsed. They nestle in furrow-like depressions of the leaf. (B) Histochemical staining of a cross-section using aqueous methylene blue/azure II and the PAS reaction. It shows the morphology of the sessile glands: glandular cells (GC); endodermal cell (EC); basal cells (BC, asterisks); leaf epidermis (LE); and mesophyll cells (MC). (C) Median vertical section of glands in TEM after freeze fixation. Arrowheads point to the cutinization of the cell walls. (D) Top view, horizontal section of the glandular head in TEM after freeze-fixation.

Sessile glandular trichomes are located in depressions of the epidermis, often in a row, and only slightly rise above it. In the mature leaf, new glands are formed alongside fully developed ones (Figs 1 and 7). Mature glands consist of two basal cells, one endodermal cell and a large head formed of eight terminal secretory cells (Fig. 7).

#### Secretory cells.

**Fig. 8.**
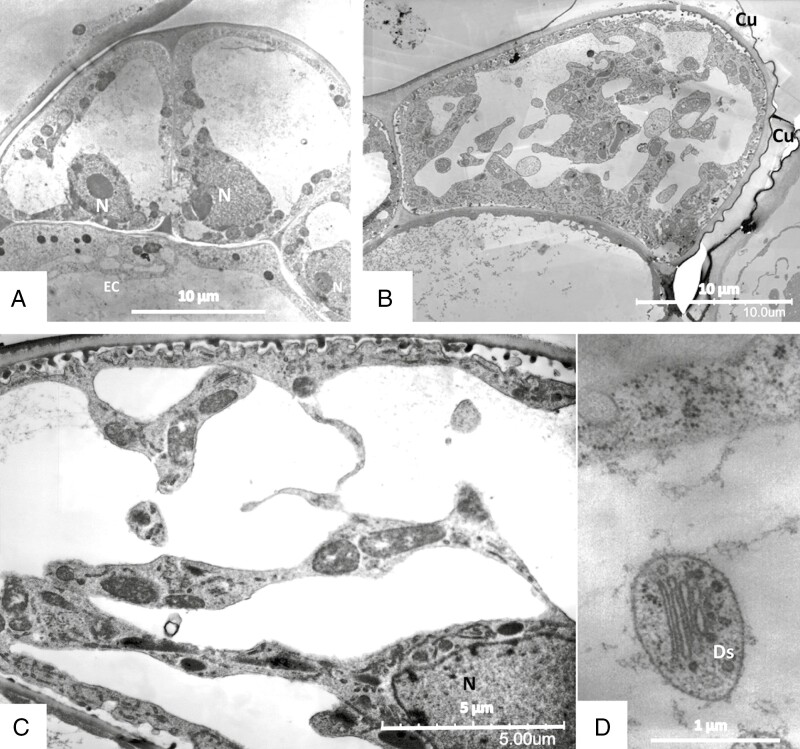
Sessile glandular trichomes. TEM of secretory cells after freeze-fixation (A, D) and chemical fixation (B, C). The cuticle (Cu) is detached from the cell wall (B). The cell wall has invaginations that are stained darker than the primary and secondary cell walls (C). The cytoplasm clusters around the nucleus (N), often in the area neighbouring the endodermal cell (EC). The central vacuole is traversed by cytoplasmic strands containing organelles, the same as in the parietal cytoplasm. (D) Cross-section through a cytoplasmic strand containing a dictyosome (Ds).

**Fig. 9.**
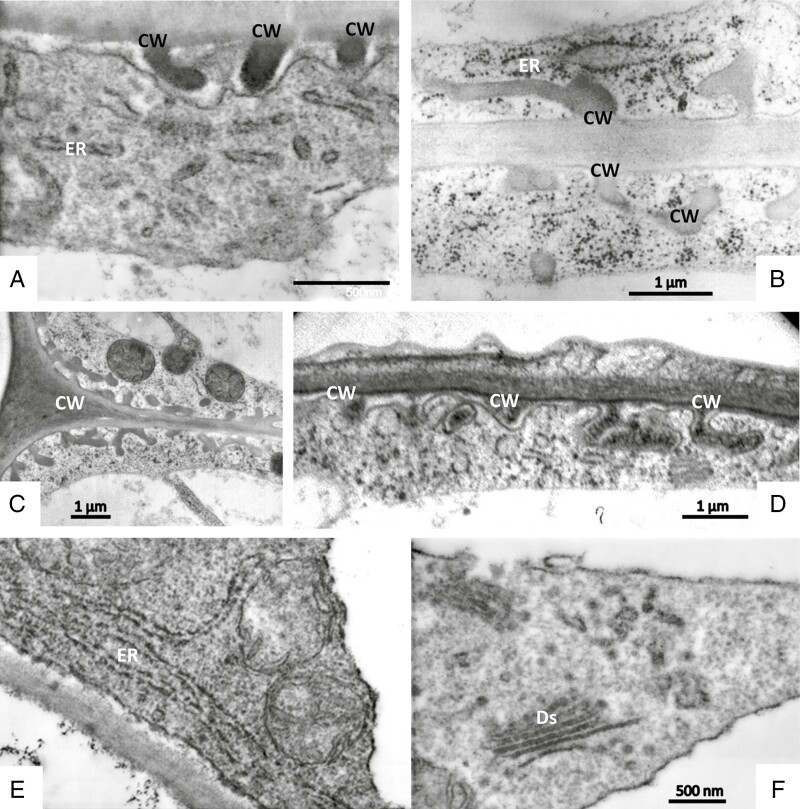
Sessile glandular trichomes. TEM of secretory cells after chemical fixation (A, E) and freeze-fixation (B–D, F). The cell wall (CW) forms invaginations; after chemical fixation they appear darker-stained than the cell wall proper (A). In freeze-fixed cells, the invaginations have an appearance similar to that of the main cell wall (B, C) or contain darkly stained substance (D). A cuticle covers the outer surface of the cell wall (D). The ER is bordered by ribosomes and binds closely to cell wall invaginations (B, E). Golgi bodies (Ds) form small smooth and coated vesicles (F).

**Fig. 10.**
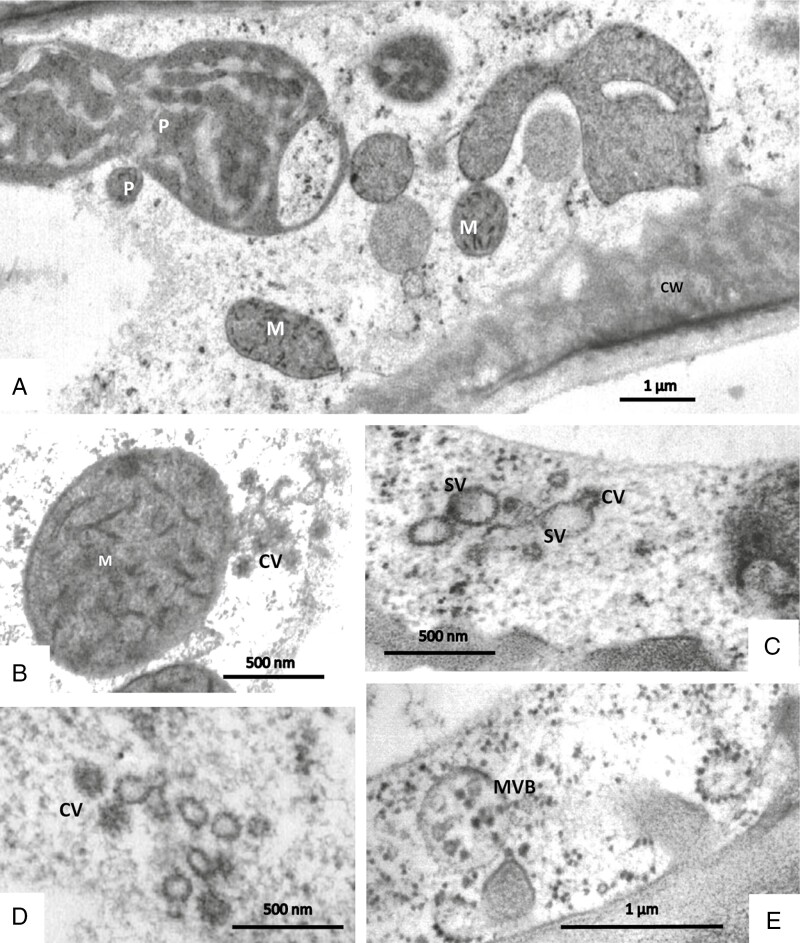
Secretory cells of sessile glandular trichomes in TEM after freeze-fixation. (A) Plastids (P) and mitochondria (M) are polymorphic. (B) Mitochondria fuse with coated vesicles (CV). (C, D) Coated vesicles fuse with smooth vesicles (SV). (E) Multivesicular bodies (MVB) occur in the parietal and central cytoplasm.

**Fig. 11.**
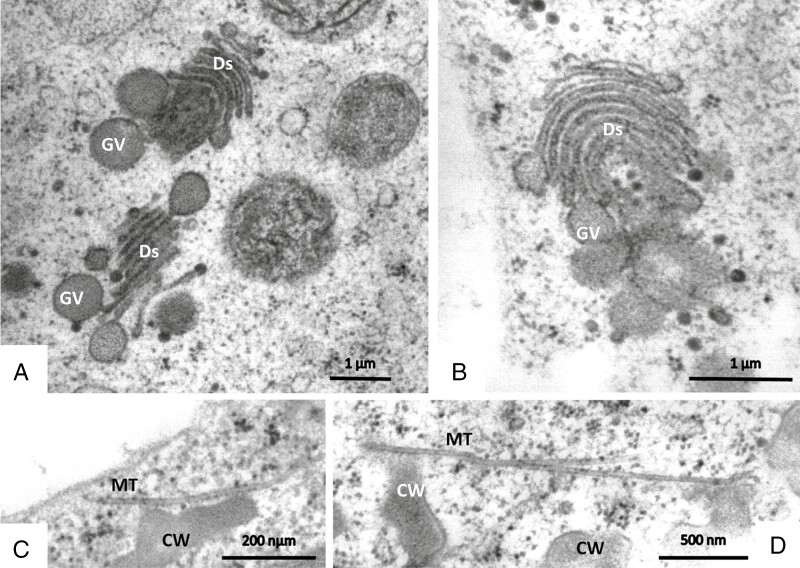
Immature secretory cells of sessile glandular trichomes in TEM after freeze-fixation. (A, B) Active Golgi bodies (Ds) produce vesicles (GV) with homogeneous greyish content of the same density as is seen in the later invaginations of the cell walls. (C, D) Microtubules (MT) occur mainly in the periphery of the cells close to the cell wall invaginations (CW), suggesting their participation in cell wall formation.

Secretory terminal cells contain a central vacuole that is surrounded by a thin layer of parietal cytoplasm containing organelles (Figs 8 and 9). Most of the cytoplasm is concentrated towards their base. The lobated nucleus is often localized, surrounded by numerous mitochondria and plastids. Transvacuolar strands traverse the central vacuole; they also contain organelles such as mitochondria and dictyosomes. These transvacuolar strands become visible in both freeze-substituted and chemically fixed cells. Plastids (leucoplasts with dense matrix) occur near the nucleus and in the basal part of the cells. They have poorly developed inner membranes and may contain small starch grains. There are dictyosomes and small vesicles, which have a spiny proteinaceous coat resembling clathrin (Figs 8D, 9F and 10C, D). They are in intimate contact with organelles such as mitochondria (Fig. 10B). Multivesicular bodies are observed near the nucleus and in the parietal cytoplasm (Fig. 10E). The ER is studded with ribosomes; it traverses the whole cytoplasm, but it becomes especially well seen as long parallel stacks in the periphery in contact with the plasma membrane where the cell wall has ingrowths. Microtubules occur near cell wall ingrowths as well; they are aligned in parallel or oblique to the cell wall. Microtubules are mainly observed in freeze-substituted material (Fig. 11C, D), less frequently in chemically fixed tissues.

Cells are in contact through plasmodesmata in cell walls between secretory cells as well as in walls between secretory cells and the endodermal cell. Cell walls consist of lightly stained primary and secondary material; on the inside of outer, radial and inner tangential cell walls they carry more densely stained homogeneous labyrinthine ingrowths, giving the cells the character of transfer cells. These wall invaginations are of reticular type (Fig. 9A–D). In chemically fixed material they are surrounded by an irregular electron-translucent zone between wall and plasma membrane (Figs 8B, C and 9A). In freeze-substituted tissue, however, the protuberances are in intimate contact with plasma membrane and cytoplasm (Fig. 9B–D).

In developing young glands of mature leaves, the cell wall is different: the inner face of the cell wall is smooth and carries no invaginations. In such cells, Golgi bodies form large vesicles with dark content that is possibly needed to build up the protuberances (Fig. 11).

**Fig. 12.**
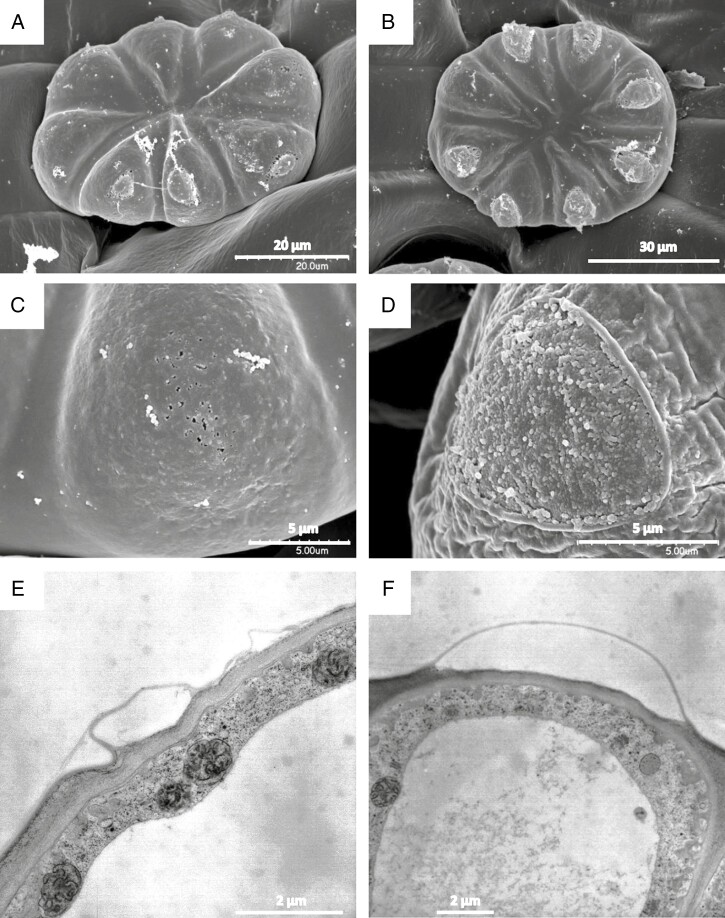
Glandular cells of sessile digestive trichomes in SEM (A–D) and in TEM after freeze-fixation (E, F). In young developing glands the cuticle contains small pores (A, C). During maturation, the cuticle opens and forms holes (B) that leave the cellulose cell wall exposed (D). TEM shows the formation of a subcuticular cavity and the detachment of the cuticle from the cell wall in cross-section (E, F).

A cuticle forms a protective layer over the head of the trichomes’ glandular cells (Fig. 8B), but observations in SEM show that each secretory cell has a small area devoid of cuticle, so that the cell wall and its microfibrils are exposed (Fig. 12B, D). Such detachment of cuticle can also be observed in TEM (Figs 8B and 12E, F). These large cuticular holes develop during maturation of the glands and are observed in material fixed by various means as well as in living cells in the light microscope.

#### Endodermal cell.

In endodermal cells the lateral cell walls are heavily impregnated with cutin; this prevents apoplastic transport and indicates their function as barrier cells. The cutinization of the lateral walls is continuous with the cuticle over the adjacent epidermal cells (Fig. 7B, C). Plasmodesmata occur in the transverse walls between the endodermal cell and the secretory cell as well as in the transverse walls between the endodermal cell and the basal cells. Plasmodesmata may be branched.

**Fig. 13.**
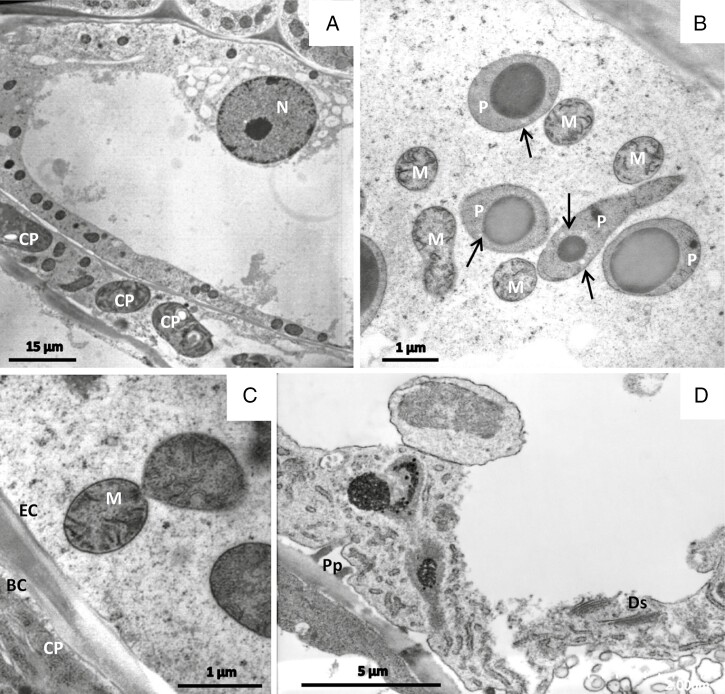
Endodermal cell (EC) of sessile glands after freeze-fixation. (A) Median vertical section shows the nucleus (N) in a cytoplasmic pocket that is filled with empty vesicles and small vacuoles. Plastids (P) with only a few small starch grains (arrows) contain large round osmiophilic bodies (B). (C) Mitochondria (M) have constrictions, probably due to division. The neighbouring basal cell (BC) has chloroplasts (CP). (D) Endodermal cell after chemical fixation. Ds, dictyosome. Note the periplasmic space (Pp) between cell wall invaginations and plasma membrane.

The cells have a large central vacuole. In addition, the parietal cytoplasm contains a net of small vacuoles which seem to accumulate in particular near the nucleus. They become especially visible in freeze-fixed cells (Fig. 13). The nucleus is located near the transverse cell walls neighbouring the secretory cells or the basal cell. The cytoplasm contains mitochondria, profiles of rough ER, multivesicular bodies and microbodies. Distinctive features are specific large ramifying plastids, which contain large osmiophilic inclusions and small starch grains (Fig. 13B).

### Basal cells and cells of the leaf epidermis and mesophyll

**Fig. 14.**
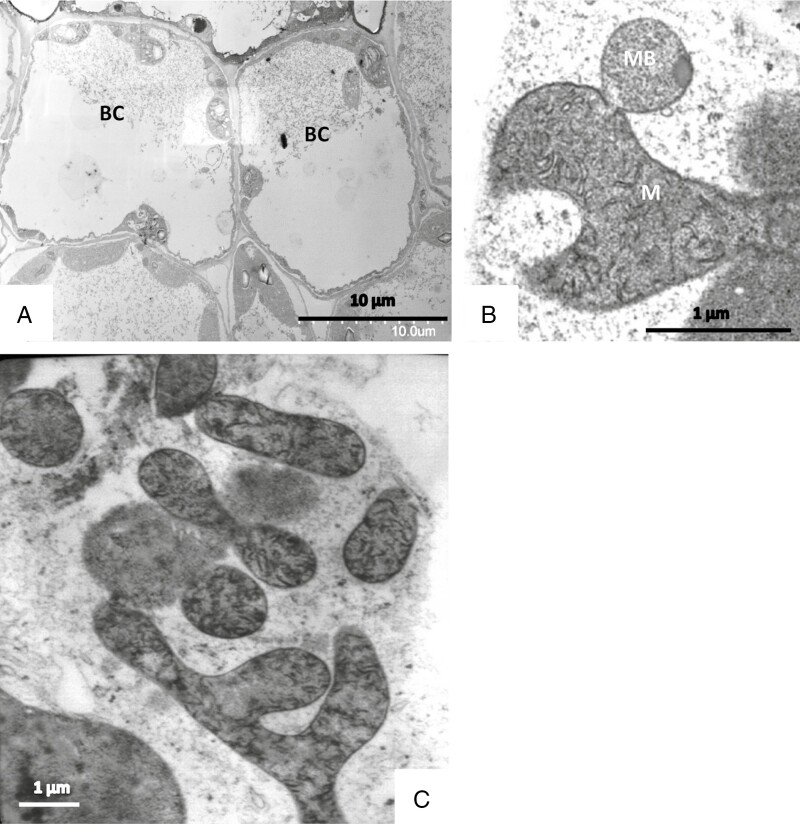
Basal cells of sessile glands in TEM after chemical fixation (A). Freeze-fixation shows (B) the intensive contacts between mitochondria (M) and microbodies (MB) and (C) the amoeboid ramifications of mitochondria.

Basal cells underlie both types of trichomes and are submerged in the leaf epidermis. They have thin tangential cell walls and provide the contact of the glandular trichomes to the cells of the leaf epidermis and mesophyll (Fig. 14). They are highly vacuolated. The parietal cytoplasm forms only a thin peripheral layer and contains the nucleus, usually near the transverse cell wall bordering the endodermal cell, and the organelles: mitochondria of amoeboid form with well-developed cristae, microbodies, and chloroplasts containing starch grains.

Neighbouring mesophyll cells have similar micromorphology, with the exception of rounded chloroplasts.

Epidermal cells contact the basal cells through plasmodesmata. Their cuticle is relatively thick and covered by a thin osmiophilic film that is structured into patterns of little spikes in particular over tangential walls. It forms a continuum with the endodermis of sessile trichomes and with the stalk cell of mucilage-producing trichomes. The cytoplasm is characterized by specific plastids contained inclusions.

### Information about the ultrastructure depends on the fixation techniques

**Fig. 15.**
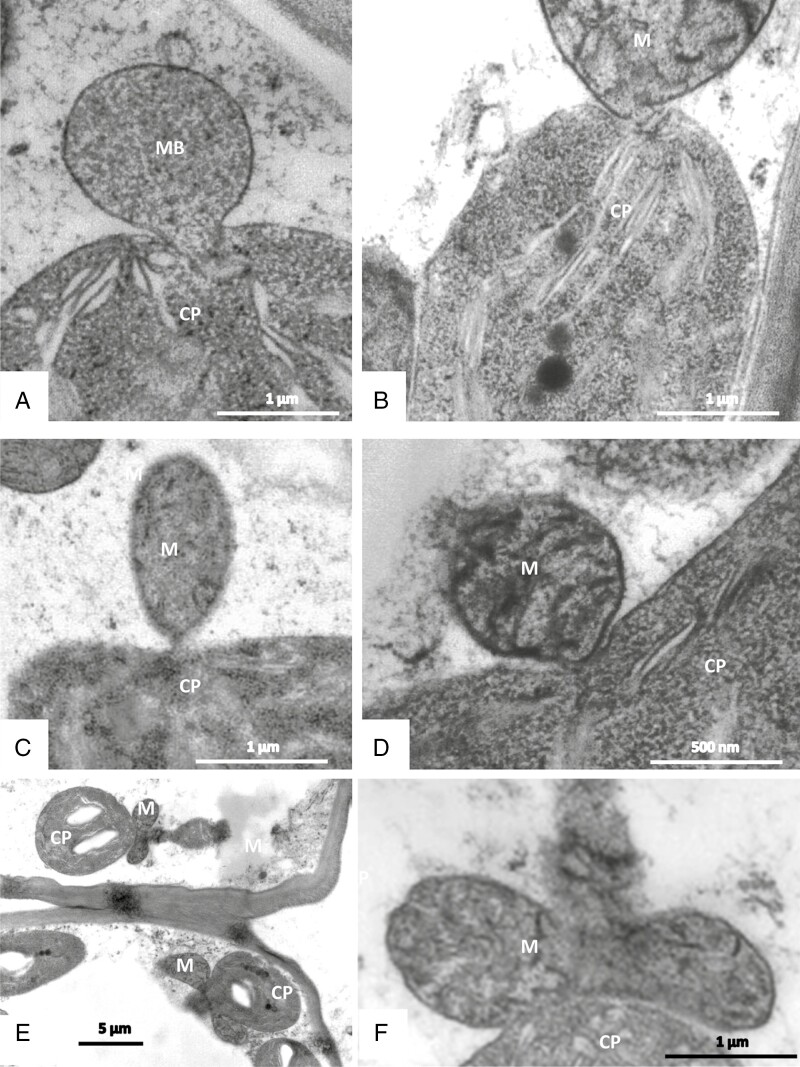
Mesophyll cells in TEM after freeze-fixation show the intimate contacts between organelles such as microbodies (MB), mitochondria (M) and chloroplasts (CP).

In all these cell types, we observed direct contact of organelles with each other: both mitochondria and plastids form clusters of associated individual organelles (Figs 14B and 15A–F), with constrictions and branches; budding of amoeboid plastids can be observed (Figs 3D and 10A). Chloroplasts and mitochondria are also in intimate contact (Fig. 15B–F). In chloroplasts, the arrangement of the thylakoid membranes appears to adapt to the contact zones of the mitochondria (Figs 14B and 15A, B). Dictyosomes and microbodies contribute to such associations (Figs 10A, B, 14B and 15A). Bundles of actin microfilaments are closely coaligned with the organelles and also with ER (Fig. 6D–H), and the ER is in close contact with the organelles (Fig. 6E).

Such associations of organelles and the cytoskeleton become mainly visible after cryo-preparation: the cytoplasm and its organelles are well preserved after freeze-fixation and freeze-substitution with minimal ice crystal damage, despite the challenge that the depth of freezing might be >100 µm. In chemically fixed cells, on the contrary, organelles appear as individuals in the conventional way that we know them, and elements of the cytoskeleton appear less frequently.

## DISCUSSION

This study presents the first detailed ultrastructural analysis of glandular trichomes of *Byblis liniflora* (Byblidaceae), a carnivorous plant that has developed sticky flypaper traps. Such adhesive traps have evolved several times within various clades of carnivorous plants, but the physiology of the glandular apparatus is remarkably similar even in unrelated taxa. Well known are the genera *Drosera* and *Drosophyllum*, where emergences from the leaf blade carry glandular cells (summarized e.g. in Juniper *et al*., 1989). In *Pinguicula* (Lentibulariaceae) and *Ibicella* (Martyniaceae), glandular trichomes produce either sticky trapping mucilage or digestive enzymes. The mucilage-producing glandular trichomes resemble closely those of *Byblis* in morphology, and their sessile glands are alike; for the understanding of *Byblis* functions, valuable information is drawn from the comparison with *Pinguicula* (Heslop-Harrison and Heslop-Harrison, 1981; Vassilyev and Muravnik, 1988; Juniper *et al*., 1989; Lang *et al.*, 1995; Adlassnig *et al.*, 2010; Ellison and Adamec, 2018; Muravnik, 2020). From anatomical and histochemical analysis, the functions of enzyme production were attributed to the various types of glands, but studies regarding the ultrastructure of the secretory apparatus are rare; in *Drosera* glandular cells, Lichtscheidl *et al*. (1990, 2021) showed the importance of good preservation by cryofixation for the study of gland physiology. We therefore made examinations with conventional chemical fixation and combined them with results from the freeze-fixed ultrastructure of the various types of cells in *Byblis* glandular trichomes in order to understand the cellular physiology of the carnivorous syndrome of these plants.

### Production and secretion of sticky mucilage and digestive enzymes

The terminal glandular cells of the trichomes are responsible for both production and secretion of substances. The synthesis of polysaccharides and other secretions in the glandular cells is energy-intensive; in *Drosera prolifera*, Adamec (2010) compared the dark respiration rate of the glands with that of the photosynthetic leaf lamina and found it more than seven times higher in the glands. These intensive metabolic processes might explain why we find a great number of mitochondria and in addition starch grains inside the leucoplasts; they provide energy, and probably also the carbohydrates that are needed for the production of the mucilage. Similar suggestions about the function of mitochondria and starch grains in oil-producing glandular cells of *Orobanche picridis* were reported by Konarska and Chmielewski (2020). In addition, all kinds of precursors and metabolites from the leaf mesophyll are needed and must be transported to the secretory cells. This is only possible through symplastic pathways and explains the presence of numerous plasmodesmata in the periclinal cell walls of endodermis and stalk cells; the cutinization of the side walls of these cells prevents any apoplastic transport (e.g. Fahn, 1988; Ascencao and Pais, 1998).

The unicellular stalks of mucilage glands easily loose turgor and collapse; such behaviour might result in the withdrawal of the prey towards the leaf surface, as was observed recently by Poppinga *et al.* (2022) and had been described also for the morphologically very similar mucilage-producing trichomes of *Pinguicula* (Heslop-Harrison, 1970).

Sticky mucilage is not specific for carnivorous flypaper traps, but serves a variety of purposes, such as nutrient provision and acquisition, adhesion and protection (reviewed recently by Galloway *et al*., 2020); it is produced and secreted by a variety of tissues and cells. In *Byblis* it consists of carbohydrates and is secreted by stalked trichomes in order to attract, adhere and retain possible prey (Lloyd, 1942; Adlassnig *et al*., 2010). We hypothesized that it is produced by the Golgi apparatus of the glandular cells, in accordance with observations from other carnivorous plants (reviewed by Juniper *et al*., 1989; Ellis and Adamec, 2018), and indeed dictyosomes produce large vesicles containing a greyish homogeneous substance. In freeze-fixed cells we can see how small vesicles of this kind fuse and form large balloons the size of the whole Golgi body; fast-freeze fixation makes visible such obviously short coalitions and thereby proves advantageous over chemical fixation. In all our pictures we observed that active Golgi bodies are not in a close spatial relationship with the ER, suggesting that polysaccharides are the main component of the final secretion.

Digestive enzymes are needed in carnivorous plants to degrade the organic compounds of the prey, and accordingly various kinds of enzymes have been detected, such as esterases, proteases, nucleases, phosphatases and others (reviewed recently by Ravee *et al*., 2018; Freund *et al.*, 2022). Based on the observations of Heslop-Harrison (2004) in the morphologically similar *Pinguicula*, their biosynthesis is usually attributed to the activities of rough ER (e.g. Juniper *et al*., 1989), and accordingly we found in the terminal secretory cells of sessile glands numerous profiles of rough ER, whereas dictyosomes appeared not active in polysaccharide production, with only tiny vesicles with a smooth or proteinaceous coat attached to their cisternae. It seems that its activity concerns the transport of hydrolytic enzymes (Vassilyev, 2005). In early developmental stages of secretory cells, however, large carbohydrate-containing vesicles were formed, which we suppose build the cell wall invaginations; this demonstrates the dual function of dictyosomes: their role in cell wall formation and their role in formation of trapping mucilage (Browning and Gunning, 1977).

An extended ‘leucoplastidome’, i.e. clusters of interconnected leucoplasts, is a conspicuous feature of secretory and endodermal cells, but possible functions can only be hypothesized. Leucoplasts in *Byblis* are most probably unable to photosynthesize, but they contain small starch grains, suggesting that they participate in the provision of metabolic energy and probably also deliver precursors for secretions. In addition, leucoplasts contain osmiophilic droplets that can fill more than half of the organelle. Leucoplasts thus resemble the secretoplasts of resin-producing stem ducts described by Joel and Fahn (1980). It has been suggested that the plastoglobuli could also maintain the shape of the lipid bodies and prevent their coalescence (Vidi *et al*., 2007). Leucoplasts with varied shapes are a common feature of glandular cells of carnivorous plant glands (e.g. Vogel, 1960; Schnepf, 1961; Heslop-Harrison, 1975; Robins and Juniper, 1980; Muravnik and Ivanova, 2002, 2003, 2004*a*, *b*; Muravnik, 2008). In carnivorous plants about 170 metabolites that have functional roles in carnivory have been found (Hatcher *et al*., 2020). Juniper *et al*. (1989) drew the conclusion from the similarity with secretoplasts that leucoplasts in carnivorous plant glands might produce volatile compounds that could lure and attract animals. This hypothesis was confirmed by ultrastructural studies linking the presence and activity of plastids to the production or secretion of various organic compounds (e.g. anthocyanins, phenolics, naphthoquinones) in Droseraceae (Muravnik and Ivanova, 2002, 2003, 2004*a*, *b*; Muravnik, 2008; see also Muravnik, 2020; Wójciak *et al*., 2023) and also by the observation that many carnivorous plant traps produce volatile substances, which can be olfactory signals for luring prey, e.g. *Sarracenia* (Miles *et al*., 1975; Jürgens *et al*., 2009; Dupont *et al*., 2023), *Heliamphora* (Płachno *et al*., 2007; Fleischmann and McPherson, 2010), *Nepenthes* (Moran, 1996; Di Giusto *et al*., 2008), *Dionaea* (Kreuzwieser *et al.*, 2014) and *Drosophyllum* (Lloyd, 1942; Ojeda *et al*., 2021). In *Drosera* volatile compounds were detected that are specific for either flowers or trapping leaves in order to protect pollinators from being trapped (El-Sayed *et al*., 2016). *Drosera* species that have scented leaves have completely non-scented flowers (Fleischmann, 2016). Further research is required to understand if *Byblis* has developed similar mechanisms to deal with pollinator–prey conflicts. On the other hand, organic compounds produced with the participation of plastids in carnivorous plants may have a role in defence against pathogens (some are fungicidal and bactericidal) and protect against animals eating the traps (Hatcher *et al*., 2020; Muravnik, 2020; Wójciak *et al*., 2023).

Export of the glandular secretions starts at the plasmalemma; the labyrinthine invaginations of the cell walls are built up during maturation of the glandular cells and increase the surface area between the plasma membrane and cell wall (Pate and Gunning, 1972). Gland exudates are further released through the cell wall and cuticle of the glandular head cells. In several descriptions, mucilage is suggested to be stored in a periplasmic space between the plasma and the cell wall before it is released; perhaps digestive enzymes could be stored and released in a similar way.

In mucilage-producing glandular cells sticky carbohydrates are stored in the extracellular space in cell walls, probably in holes developed in mature cells.

### *Cuticle discontinuities in* Byblis

Traps of carnivorous plants are mostly of leaf origin (but inflorescences may also be trap organs); they are thus covered by cuticle, which serves as a hydrophobic and protective barrier. Due to the variety of glandular organs, trap types and probably also prey specialization, carnivorous plants have developed cuticles of different structures. Common to all is that they are permeable and contain discontinuities over the glandular cells, so that the glands can secrete and absorb substances. Cuticle discontinuities are easily detected using vital dyes (e.g. an aqueous solution of neutral red or methylene blue; Lloyd, 1942; Joel and Juniper, 1982; Joel *et al*., 1983; Anderson, 2005; Płachno *et al*., 2005; Adlassnig *et al*., 2012), thanks to which it is possible to track when discontinuities appear during maturation, i.e. when the gland cells are ready for secretion and/or absorption. An exception is the cuticle of the digestive glands of *Dionaea muscipula*, where discontinuities (cuticular gaps) appear after protein stimulation (Joel *et al*., 1983). In *Byblis gigantea*, Lloyd (1942), using methylene blue, proved the occurrence of a permeable cuticle over terminal cells of both sessile and stalked trichomes. He described cuticular pores in terminal cells of stalked trichomes as ‘large oval openings arranged in a circle about and some distance away from the centre of the capital’ (Lloyd, 1942, p. 97). In this species, pores were also observed in sessile trichomes by Poppinga *et al.* (2022) using SEM. According to these authors mucus residues are visible in pores; however, they did not describe the structure of these pores. According to Li *et al.* (2023), in *Byblis guehoi* pores lack hydrophilic components such as cellulose and pectin and look like a hole. However, we have some doubts as to the correct interpretation of the results by these authors, especially since some of the images seem to indicate that the signal from autofluorescence may have been confused by the authors with the results of staining. Our ultrastructural results clearly indicate that the cell wall is still present after the formation of the cuticular discontinuities. Our studies indicate that cuticular ‘pores’ in glandular cells in *Byblis* take the form of large areas devoid of cuticle, in particular a cuticularized layer that becomes detached during trichome maturation, and that these areas become large by rupturing of the cuticle. Further research is needed to determine how the cutinized layer is developed in cells of these trichomes. Exposure of a large area of the cell wall results in facilitation of both secretion and absorption of water-soluble substances. Due to their mode of formation and size, cuticular discontinuities in *Byblis* cannot be classified as pores, as they had been described in *Drosera* (Williams and Pickard, 1969, 1974; Ragetli *et al.*, 1972; Juniper *et al*., 1989), *Genlisea* (Płachno *et al*., 2005) and *Roridula* (Anderson 2005). We propose for cuticular discontinuities in *Byblis* the term ‘cuticular holes’. Formation of a permeable cuticle by rupturing occurs also in trichomes of *Utricularia* traps. These trichomes produce mucilage and velum (Fineran, 1985; Heide-Jørgensen, 1991; Płachno *et al*., 2019). According to Heide-Jørgensen (1991), in secretory cells of these trichomes no cuticular layer is formed, and the velum is a slightly modified procuticle. He suggested that at maturation it is by homology a cuticle proper. However, despite the fact that both *Utricularia* and *Byblis* have a ruptured cuticle, there are distinct differences. The main one is that in *Byblis* the rupture of cuticle occurs only in one place in the cell, while in *Utricularia* the whole top part of the cell is devoid of cuticle. Therefore, we consider the formation of cuticular discontinuities in glandular cells in *Byblis* to be exceptional and unique among carnivorous plants.

Transport of solutes between leaf and glands is provided through the single endodermal (endodermoid) cell, a feature so far found in every kind of carnivorous plant. Radial walls are heavily cutinized or suberized, especially in the region right underneath the secretory head cells, resembling the Casparian strip of the root endodermis. They thus are impermeable for the transport of substances between leaf and secretory cells through the apoplast, as was shown for *Pinguicula* (Heslop-Harrison and Knox, 1971; Heslop-Harrison, 1976), but solutes take the symplastic pathway; accordingly, in *Byblis* the tangential walls of the endodermal cell form numerous plasmodesmata, either simple or branched, and also partly grouped.

Energy in the secretory cells is most probably also supplied by solutes from the leaf: basal cells were also described as reservoir cells (Heslop-Harrison and Heslop-Harrison, 1981) and suggested in *Pinguicula* to play an important role in the secretory activity of the glands (Heslop-Harrison, 1976), especially in the accumulation and distribution of solutes during transport into and out of the gland, and in the provision of energy.

The stalk cells of mucilage-producing trichomes have thick cell walls and a thick cuticle, which become especially conspicuous underneath the endodermal cell and appear to carry the glandular head upright. Oblique or spiraliform orientation of the cellulose microfibrils becomes visible in TEM and explains the twisting and kinking motions described by Allen (2019) and Poppinga *et al*. (2022). These chemonastic movements were suggested to bring the prey into close contact with the sessile glands and their digestive enzymes.

### What information can be drawn faithfully from TEM?

Evidence about the ultrastructure of glandular cells from carnivorous plants comes mainly from conventional EM, where the tissue is fixed with aldehydes and osmium; valuable information has been drawn from this research. Nevertheless, chemical fixation is suspected to lead to severe changes in the cytoplasm and its organelles (e.g. Kuo, 2007). This becomes especially critical in plant cells that are impregnated by cutin and other waxes in their cell walls: the cuticle prevents fast exchange of solutes through the cell walls, and the osmotic values are changed. A special example in *Byblis* is provided by the long stalk cells of mucilage trichomes (Figs 2 and 3): during fixation for histochemistry and also during freeze-substitution after freeze-fixation, the turgor is lost, the cell walls collapse, and the cells wrinkle and shrink. We hypothesize that such turgor loss is also the reason for the twisting and shrinking of the stalked trichomes in *B. gigantea* that have been described recently (Poppinga *et al*., 2022).

Change of turgor might also be responsible for the different appearance of cell walls in digestive secretory cells: while in chemically fixed tissue a periplasmic space appears between plasma membrane and cell wall, in cells after freeze-fixation the cytoplasm closely aligns with the cell walls (Figs 5, 8 and 9). Such periplasmic space has also been described in other secretory cells, such as mucilage cells and nectary cells (Trachtenberg and Fahn, 1981; Paiva, 2016; Płachno *et al*., 2016), and it has been hypothesized that it serves for enzyme and also other secretory product storage. In consideration of the pictures yielded by freeze-fixation, we suggest that this periplasmic space in *Byblis* has appeared due to either osmotic shrinkage of the cytoplasm or due to temporary swelling followed by deswelling of the cell wall, as had been proposed already by Browning and Gunning (1977).

The slow penetration of solutes through aerial parts of the plant has an additional disadvantage: the concentration of fixatives increases only gradually and the cytoplasm has time to react to the toxic substances. In *Byblis*, for instance, the stalk cells of the mucilage-producing trichomes have highly dynamic organelles that can be observed in the light microscope moving within the peripheral cytoplasm and through cytoplasmic strands. During immersion in chemical fixatives, their movement continues for up to 10 min and only gradually slows down and stops, suggesting that the cytoskeleton has become inactivated. Large organelles such as plastids change their form and lose their associations with other organelles during this fixation period (not shown). In addition, we observe that the cytoplasm no longer spans the vacuole in the form of transvacuolar cytoplasmic strands, but retracts into the parietal cytoplasm. This leads to relocation of organelles.

In contrast, freeze-fixation works extremely fast; the cytoplasm is immobilized within a split second. The organization of the cytoplasm with vacuoles and transvacuolar cytoplasmic strands therefore remains unchanged, organelles have no time to relocate, and the associations between cytoskeleton, ER and organelles persist. Of course volumetric reconstructions from serial sections showed also in conventional fixation techniques how organelles may cluster and interconnect (e.g. Gunning and Steer, 1996); however, the frequency of such observations is much higher after quick cryofixation: in almost every kind of cell we observe organelle interactions: mitochondria and also plastids form ramified and constricted super-aggregates, and they are also in close contact with each other

For mitochondria, e.g. Foissner (2004) demonstrated in Characean algae how their shape may be vermiform, sausage-form, amoeboid or rounded and rod-like; it depends on actin microfilaments and microtubules. We conclude that the amoeboid form of mitochondria and plastids in *Byblis* depends on the cytoskeleton, and that associations of organelles might be only intermediate and short-term, so that they are loosened and lost during chemical fixation and become visible only after freeze-fixation.

### Quality of ultrastructural preservation after chemical and freeze-fixation

Where freeze-fixation was used, the different cell types normally showed only little sign of ice crystal damage: secreting cells, transfer cells and cells of the leaf all had areas that were well preserved, although in some parts of the cells, often directly adjacent, ice crystal damage occurred. In such cases, the cytoplasm was disrupted. The heavily cutinized stalk cells of the mucilage-producing trichomes were seriously distorted during the fixation and embedding procedures, similar to what was seen after chemical fixation: the long cells were no longer straight but twisted and the lateral walls had collapsed. The cytoplasm, however, was very well preserved, the plasma membrane closely aligned with the cell wall, the cytoskeletal elements became clearly visible, and associations of organelles and ER could be observed. Obviously such turgor changes did not affect the cytoplasm severely.

Some controversy exists over the need for cryoprotectants during freeze-fixation, over the length of freeze-substitution, and over the depth of good freezing quality (see discussion in Huebinger *et al*., 2016). Cryoprotectants such as glycerol might cause alterations of the osmotic relations, e.g. plasmolysis (Willison, 1976). In *Drosera* (Lichtscheidl *et al*., 2021) and also in *Byblis* described here, no cryoprotectants were needed. We relate this to the occurrence of natural cryoprotectants such as mucilage in these cells, similar to Fisher (1975): cells with naturally occurring cryoprotecting compounds had less ice crystal damage than adjacent cells. In addition, cells with dense cytoplasm and numerous ribosomes and organelles, such as the glandular cells described here, have less free water in their cytoplasm and accordingly form fewer and smaller ice crystals (Gunning, 1977).

As a problematic feature in well-preserved freeze-fixed cells we observe that the vitrified cytoplasm and matrices of mitochondria and plastids are denser, as had been described already in the early days of cryofixation, e.g. by Kiss *et al.* (1990). Also, the plasma membrane and internal membranes are smoother and have only little or no contrast (e.g. Figs 5A and 10A); it may therefore be difficult to address the identity of organelles. On the contrary, where slight ice crystal formation occurs, membranes obtain good contrast and the matrixes of organelles are a little extracted (Fig. 5D, E, Supplementary Data Fig. 2).

## Conclusions

Our research shows the ultrastructure of the cytoplasmic specialization of individual cell types in the glands of *B. liniflora*, which is reflected in the development of cell walls, cuticles and the differentiation of organelles. We show the advantages of cryo methods in preserving organelles, their associations with each other, and their interactions with the cytoskeleton. In addition, the close association of the plasma membrane with the invaginations of the cell wall becomes visible. The cuticle of digestive glands forms unique cuticular holes.

The next stage of the study should be to analyse changes in *Byblis* glands after feeding using cryo methods. In addition, it would be worthwhile to extend the study to the cell wall structure of gland cells in terms of differences in composition.

## SUPPLEMENTARY DATA

Supplementary data are available *at Annals of Botany* online and consist of the following.

Figure S1: stalk cells of mucilage-producing glands. Figure S2: slight ice crystal damage in a secretory cell of a digestive gland after freeze-fixation.

mcae173_suppl_Supplementary_Figures_1

mcae173_suppl_Supplementary_Figures_2
