# Supplementary figures and images for: Cyto-architecture of Byblis glands and leaf cells based on freeze-substitution and conventional TEM
Source: Ann Bot. 2024 Sep 27;135(3):463–82. doi: 10.1093/aob/mcae173 (PMC11897603; doi:10.1093/aob/mcae173)

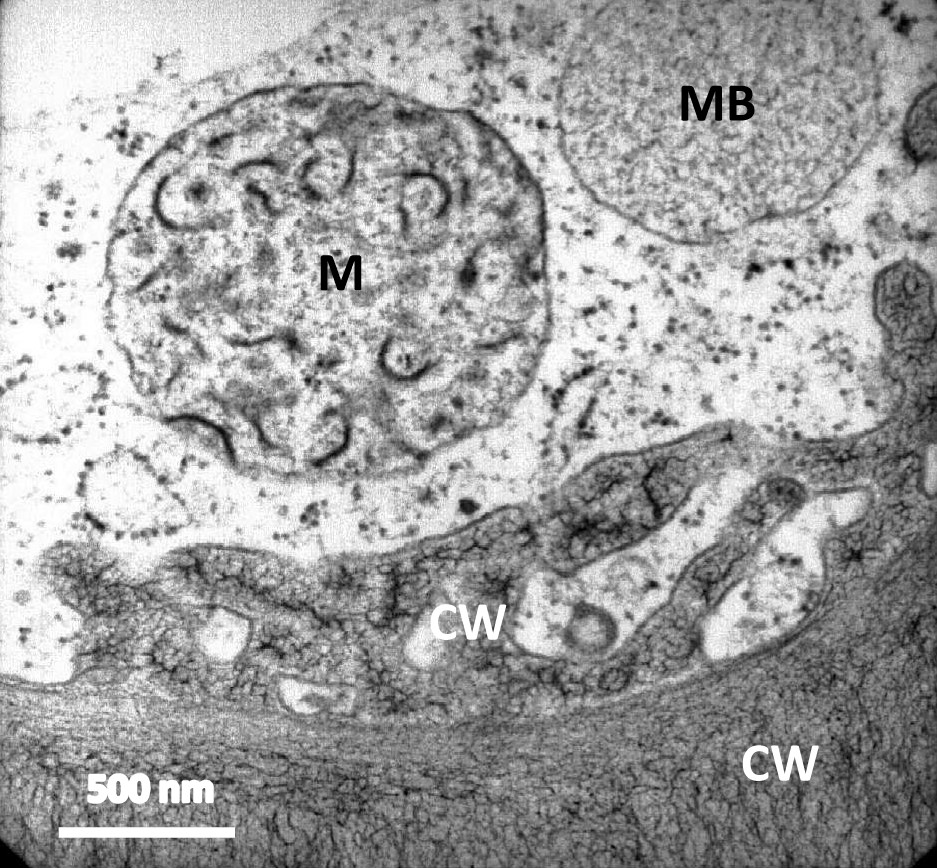

Supplement: mcae173_suppl_Supplementary_Figures_1 [file mcae173_suppl_supplementary_figures_1.jpeg]

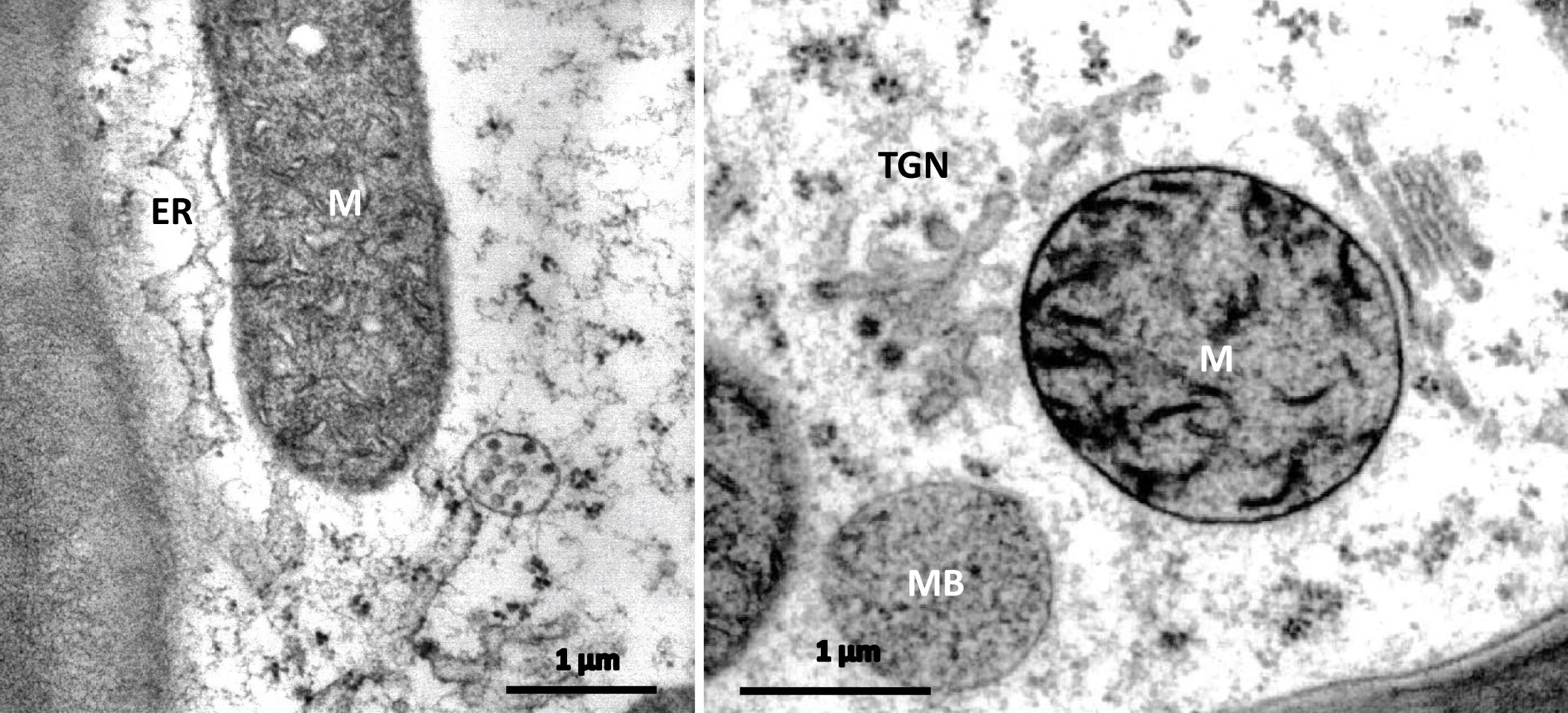

Supplement: mcae173_suppl_Supplementary_Figures_2 [file mcae173_suppl_supplementary_figures_2.jpeg]
